# Investigation and Control of Anthrax Outbreak at the Human–Animal Interface, Bhutan, 2010

**DOI:** 10.3201/eid2009.140181

**Published:** 2014-09

**Authors:** Nirmal K. Thapa, Tenzin, Karma Wangdi, Tshering Dorji, Migma, Jambay Dorjee, Chung K. Marston, Alex R. Hoffmaster

**Affiliations:** National Centre for Animal Health, Thimphu, Bhutan (N.K. Thapa, Tenzin, Migma, J. Dorjee);; Ministry of Health, Thimphu (K. Wangdi, T. Dorji);; Centers for Disease Control and Prevention, Atlanta, Georgia, USA (C.K. Marston, A.R. Hoffmaster)

**Keywords:** anthrax, outbreak, epidemiology, control, human–animal interface, Bhutan, cutaneous anthrax, Bacillus anthracis, vaccination, isolation, ruminants, transmission, spread, livestock, humans, control measures, bacteria, zoonoses

## Abstract

In 2010, we investigated anthrax outbreak in Bhutan. A total of 43 domestic animals died, and cutaneous anthrax developed in 9 persons, and 1 died. All affected persons had contact with the carcasses of infected animals. Comprehensive preparedness and response guidelines are needed to increase public awareness of anthrax in Bhutan.

Anthrax, an acute infectious disease caused by infection with *Bacillus anthracis*, can affect almost all warm-blooded animals, including humans (*1*). Animals become infected through contact with soilborne *B. anthracis* spores; humans become infected only incidentally through contact with diseased animals or with the carcasses or by-products of diseased animals (*1*). Anthrax is widespread. Sporadic outbreaks and epizootics occur among livestock and wild animals in the United States, Canada, and southern and eastern Europe, and outbreaks at the animal–human interface are reported from countries in Africa, the Middle East, and Asia (*1*–*7*). In southern Asia, anthrax is highly endemic in India and Bangladesh, and frequent outbreaks and cases are reported among animals and humans (*6*–*8*).

In Bhutan, sporadic anthrax outbreaks occur annually among animals, posing health risks to persons who come into contact with the infected animals (*9*). We present the findings of an epidemiologic investigation of a major anthrax outbreak that occurred at the human–animal interface in a remote area of central Bhutan.

## The Study

During July–September 2010, an outbreak of anthrax occurred among animals in Kaktong, a remote village in Zhemgang District in central Bhutan. The outbreak later spread to 8 neighboring villages, where humans also became infected. The outbreak began after a period of heavy rainfall, which may have brought spores to the soil surface, where they could be ingested by ruminants grazing in the area.

A multisectoral team from animal and public health offices in Bhutan visited the outbreak area to investigate and to establish a control program. A case of anthrax was suspected if an animal had signs or symptoms of infection (e.g., sudden death, bloated carcass, bleeding of unclotted blood from natural orifices); a case of anthrax was confirmed if rod-shaped bacilli were found by blood smear examination. Additional samples from animals with positive blood smears were referred to the US Centers for Disease Control and Prevention (Atlanta, GA, USA) for culture and isolate characterization. *B. anthracis* was isolated from 3 samples (2 ear tips and 1 nasal swab) from 3 cattle (from 3 separate villages).

**Figure 1:**
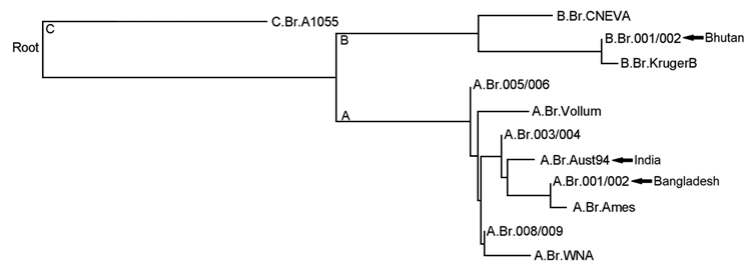
Phylogeny of major *Bacillus anthracis* groups as determined by using canonical single nucleotide polymorphisms as described by Van Ert et al. (*12*). Arrows indicate the lineages/groups of genotyped *B. anthracis* isolates from India (*12*), Bangladesh (*11*,*13*), and Bhutan.

All isolates were characterized by multilocus variable-number tandem repeat analysis, and 1 isolate was analyzed by whole-genome sequencing and single-nucleotide polymorphism analysis (*10*,*11*). Other strains from nearby Bangladesh and India were recently characterized and belong to the more widely dispersed A lineage. However, isolates from the Bhutan outbreak were found to be part of the multilocus variable-number tandem repeat analysis B1 lineage (genotype 83) and canonical single-nucleotide polymorphism subgroup B.Br.001/002 (Figure 1) (*10*–*13*). The B lineage is less widespread and primarily associated with South Africa, but it has been reported in parts of the United States, Europe, and Asia, including the Caucasus region in a recent report (*10*–*14*). The team in Bhutan investigated the mode of *B. anthracis* transmission and spread among livestock and humans in outbreak areas and implemented control measures.

**Figure 2:**
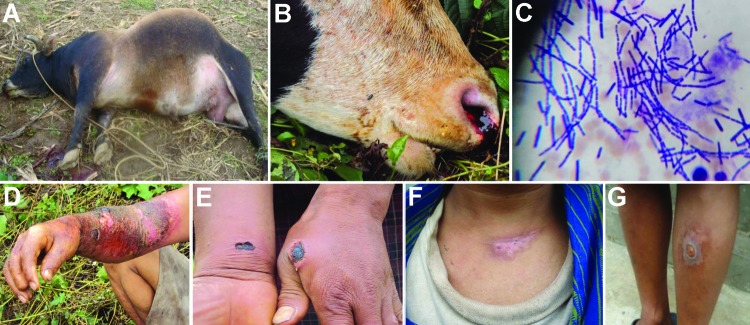
Signs of anthrax in infected animals, Bhutan, 2010. A) The carcass of an affected bull, showing bloating. B) Bleeding of unclotted blood from a cow’s nostril. C) Rod-shaped *Bacillus anthracis* bacilli from 1 of the infected animals.

In Kaktong, the index village, a cow had suddenly died after a brief illness; the animal exhibited bleeding of unclotted blood from nostrils, and its carcass was bloated (Figure 2) (*1*). The owner of the affected herd had opened the carcass and dressed the meat, which he shared or sold within the village for human consumption. Transportation of infected meat to neighboring villages resulted in the spread of disease and death among animal herds in 8 other villages; like the index animal, the animals that died were dressed out for human consumption. In some instances, horses that were used to carry contaminated meat became infected and died of anthrax. During July–September, a total of 43 animals in 9 villages died: 25 cattle, 8 horses, 4 pigs, and 6 cats. The infected cats were possibly exposed to *B. anthracis* through the ingestion of meat from infected carcasses. The infected pigs were fed with *B. anthracis*–contaminated kitchen waste.

**Figure 3:**
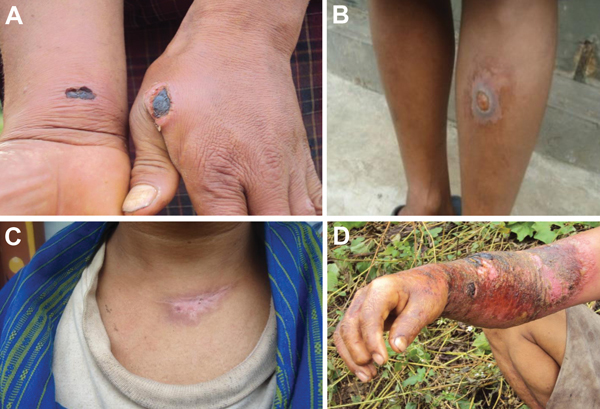
Cutaneous anthrax with typical black eschars on the hand and wrist (A), leg (B), and neck (scar) (C); and severe inflammation of the arm (D) of persons who had contact with *Bacillus anthracis*–infected animals and carcasses, Bhutan 2010.

The investigation showed that within 1 week of exposure, skin lesions developed on 9 humans (8 male; 1 female) who handled and dressed the animal carcasses. The lesions were black eschars, typical of anthrax, and occurred on the patients’ necks, fingers, arms, feet, or cheeks (Figure 3). Of the 9 persons with cutaneous anthrax, 1 (the female villager) died. Symptoms consistent with gastrointestinal anthrax, including abdominal cramps, vomiting, and respiratory distress, developed in this person after she ingested contaminated meat. A detailed investigation of the case was not conducted because of the remote location of the village.

To stop the spread of disease, animal and public health authorities initiated various prevention and control measures: a campaign to create awareness among villagers and students; ring vaccination of cattle against anthrax (≈445 animals in 11 villages); treatment of sick animals with antimicrobial drugs ; disposal of carcasses in deep burial pits; recall, collection, and disposal into burial pits of all potentially infected meat and hides from cattle that died of suspected or confirmed anthrax; and monitoring and treatment of persons in whom cutaneous anthrax developed. These control measures eventually contained the outbreak.

In the remote villages of Bhutan, meat from dead animals is usually consumed by the villagers because they live in poverty and lack slaughterhouse facilities and education regarding diseases that might harm them. As in other remote villages, the farmers in Kaktong and neighboring villages affected by this outbreak were unaware of anthrax in animals and of the public health implications of this disease. Our investigation showed that all affected persons had handled and/or consumed meat from animals with suspected or confirmed anthrax. While we were conducting the outbreak investigation and control programs, we held an education meeting to make villagers and students aware of the risks associated with anthrax. The villagers cooperated in response activities, including the disposal of carcasses and recall and disposal of meat from the carcasses that had been kept for human consumption, and they participated in control activities, including the treatment of affected animals and ring vaccination of animals that had been in contact with infected animals.

In Bhutan, sporadic anthrax cases in animals are detected and reported every year; such cases pose risks to humans (*9*). The sudden emergence in 2010 of an anthrax outbreak in remote villages in Bhutan could be linked to heavy rainfall, which may raise *B. anthracis* spores to the soil surface, where they can be ingested by animals. Cutaneous anthrax cases similar to those reported here have been reported at the human–animal interface in other countries. For example, >6,000 anthrax cases in humans were reported in Zimbabwe in 1979 and 1980; the cases were associated with the slaughter of *B. anthracis*–infected cattle (*4*). In addition, 25 cutaneous anthrax cases occurred in humans in Paraguay in 1987 after the slaughter of a single *B. anthracis*–infected cow (*5*), and many cases of cutaneous anthrax have occurred in humans following the slaughter of sick or dead animals in India (*6*), Bangladesh (*8*), and China (*15*).

For humans, the major sources of exposure to *B. anthracis* are direct or indirect contact with infected animals or contaminated animal products. Persons at risk for exposure should be made aware of those risks and of the public health implications of zoonotic diseases such as anthrax (*1*). From this outbreak investigation and our experiences in Bhutan, we recommend the following measures for this country: development of comprehensive guidelines for anthrax surveillance among humans and animals; establishment of surveillance for anthrax hot-spot areas; and development of education programs to teach persons at high risk (e.g., butchers) about anthrax transmission, the care of skin abrasions, and disease-prevention measures, including personal hygiene practices and refusal to eat meat from dead or sick animals.
